# Can Deep Learning-Based Auto-Contouring Software Achieve Accurate Pelvic Volume Delineation in Volumetric Image-Guided Radiotherapy for Prostate Cancer? A Preliminary Multicentric Analysis

**DOI:** 10.3390/curroncol32060321

**Published:** 2025-05-30

**Authors:** Cristiano Grossi, Fernando Munoz, Ilaria Bonavero, Eulalie Joelle Tondji Ngassam, Elisabetta Garibaldi, Claudia Airaldi, Elena Celia, Daniela Nassisi, Andrea Brignoli, Elisabetta Trino, Lavinia Bianco, Silvia Leardi, Diego Bongiovanni, Chiara Valero, Maria Grazia Ruo Redda

**Affiliations:** 1 Department of Oncology, University of Turin School of Medicine, 10126 Turin, Italy; cristiano.grossi93@gmail.com (C.G.); eulaliejoelle.tondjingassam@unito.it (E.J.T.N.); 2 Department of Radiation Oncology, Umberto Parini Hospital, 11100 Aosta, Italy; ibonavero@ausl.vda.it (I.B.); egaribaldi@ausl.vda.it (E.G.); ecelia@ausl.vda.it (E.C.); abrignoli@ausl.vda.it (A.B.); 3 Department of Radiation Oncology, Mauriziano Umberto I Hospital, 10128 Turin, Italy; cairaldi@mauriziano.it (C.A.); dnassisi@mauriziano.it (D.N.); etrino@mauriziano.it (E.T.); lvbianco@mauriziano.it (L.B.); sleardi@mauriziano.it (S.L.); dott.diegobongiovanni@gmail.com (D.B.); 4 Department of Medical Physics, Mauriziano Umberto I Hospital, 10128 Turin, Italy; cvalero@mauriziano.it; 5 Department Oncology, University of Turin School of Medicine, Mauriziano Umberto I Hospital, 10128 Turin, Italy; mariagrazia.ruoredda@unito.it

**Keywords:** auto-contouring, organs at risk, deep learning, segmentation, prostate cancer, pelvic lymph nodes, Limbus^®^ Contour

## Abstract

Background: Radiotherapy (RT) is a mainstay treatment for prostate cancer (PC). Accurate delineation of organs at risk (OARs) is crucial for optimizing the therapeutic window by minimizing side effects. Manual segmentation is time-consuming and prone to inter-operator variability. This study investigates the performance of Limbus^®^ Contour^®^ (LC), a deep learning-based auto-contouring software, in delineating pelvic structures in PC patients. Methods: We evaluated LC’s performance on key structures (bowel bag, bladder, rectum, sigmoid colon, and pelvic lymph nodes) in 52 patients. We compared auto-contoured structures with those manually delineated by radiation oncologists using different metrics. Results: LC achieved good agreement for the bladder (median Dice: 0.95) and rectum (median Dice: 0.83). However, limitations were observed for the bowel bag (median Dice: 0.64) and sigmoid colon (median Dice: 0.6), with inclusion of irrelevant structures. While the median Dice for pelvic lymph nodes was acceptable (0.73), the software lacked sub-regional differentiation, limiting its applicability in certain other oncologic settings. Conclusions: LC shows promise for automating OAR delineation in prostate radiotherapy, particularly for the bladder and rectum. Improvements are needed for bowel bag, sigmoid colon, and lymph node sub-regionalization. Further validation with a broader and larger patient cohort is recommended to assess generalizability.

## 1. Introduction

Prostate cancer is the most common malignancy in the male population and consumes a significant number of resources in Radiation Oncology departments [1,2,3]. In Italy, prostate cancer accounts for over 20% of all cancers diagnosed in men over the age of 50. In 2022, there were approximately 40,500 new cases, while in 2023, the number increased to 41,100 and in 2024 it was 40,192. In total, 8200 men died because of prostate cancer in Italy in 2022 [4].

The treatment of prostate cancer must be personalized, considering the stage and aggressiveness of the disease, as well as the patient’s life expectancy and the presence of any comorbidities that may increase the risk of mortality compared to the prostate cancer itself.

Radiotherapy (RT) is a therapeutic option for the treatment of prostate cancer with curative intent [5,6,7,8,9,10,11,12]. Equally radical prostatectomy (RP), involving surgical removal of the prostate, vas deferens, and seminal vesicles (with or without lymphadenectomy), is a common treatment for prostate cancer. However, even after RP, biochemical recurrence of disease (BCR) occurs in 27–53% of patients [13,14]. Adjuvant radiotherapy (ART) following RP has demonstrated a 50% reduction in BCR risk for patients with high-risk features [15,16,17]. A recent large retrospective study further emphasizes the benefit of ART in patients with positive lymph nodes (pN1), a high Gleason score (pathological GS 8–10), and extra prostatic extension (pT3/pT4), showing a reduction in all-cause mortality rates [18]. The salvage setting after RP, where cancer recurs, has become a crucial area of research. The ARTISTIC meta-analysis, encompassing trials like RADICALS-RT, TROG 08.03/ANZUP RAVES, and GETUG-AFU 17, supports the PSA-based approach and the role of salvage radiotherapy (SRT) in patients who were previously considered candidates for ART [19,20,21,22]. The RTOG 0534 trial showed a marginal benefit of prophylactic pelvic nodal irradiation (PNRT or ENRT) [23]. Consequently, RT plays an essential role in prostate cancer management in both radical and salvage setting by delivering a targeted high dose to eradicate cancerous cells while minimizing harm to surrounding healthy tissues. This delicate balance is crucial for mitigating both short- and long-term side effects. However, a major bottleneck in the treatment planning process is the manual segmentation of target volumes and organs at risk (OARs) on Computed Tomography (CT) scans [24]. Historically, manual segmentation of structures on 3D anatomical images, typically CT scans, has been performed by clinical experts. While this ensures expert review, it is also a very time-consuming process. Literature reports mean manual segmentation times for head and neck cases ranging significantly, from 28.5 min up to 3 h, depending on the specific structures being delineated. This time-intensive nature, coupled with the inherent susceptibility of manual segmentation to inter- and intra-observer variability, has driven the increasing adoption of automatic techniques over the past decade [25].

While manual contouring of organs at risk (OARs) and clinical target volumes (CTVs) is an essential component of radiotherapy (RT) planning, its time-consuming nature and reliance on staff availability contribute substantially to RT treatment planning lead times [26].

Artificial Intelligence (AI) can provide a powerful contribution to a lot of human-dependent steps in RT, considering that human participation is a principal uncertainty source, potentially impacting on the efficacy of treatments. In particular, the main fields of applications of AI in RT are the following:

- Lesion and OAR contouring, with data derived from fusions of multimodal imaging: The accuracy of auto-segmentation is higher for structures that have a high contrast against their surrounding tissues (lung, eye, bladder), while it is lower in the case of OARs with small volumes and fuzzy boundaries (optic chiasma). In clinical practice, manual checking is necessary, with a consideration of the different reference guidelines of different institutions.
- Treatment Planning: AI can help in augmenting dose map prediction (Dose Volume Histograms (DVHs) and voxel-based dose prediction) and in supervising and guiding the optimization process, which usually requires sequential modifications of parameters such as target coverage, OAR constraints and their priorities, selecting the ones that need an update and also allowing for procedures of replanning and adaptive RT to be completed more quickly.
- Patient- and machine-specific quality assurance: The purpose of this is to ensure consistency between the medical prescription and its delivery, reducing the workload involved in measuring and analyzing doses using a phantom and in the assessment of the performances of all devices involved in RT. AI algorithms can also help in relating the spatial dose to RT outcomes, consenting prognosis predictions and prediction of the risk of side effects.

A major limitation in the use of AI in RT practice is the lack of regulation, because these systems do not have 100% accuracy, so human surveillance is essential. Furthermore, the use of AI could lead to young radiation oncologists dealing with matters beyond their level of expertise, since they have to face problems and devise solutions, particularly in checking results. This could cause issues in the advancements made within the discipline [27].

A thorough comprehension of these technologies is essential to warranting the optimal use of these tools. Advanced AI techniques, like Convolutional Neural Networks (CNNs) with high-performance predictions for complex problems, lead to the so-called “black box” problem: a lack of transparency and knowledge about the functioning of the machine learning models. This cultural barrier can dampen the faith in AI solutions held by health professionals. A technology known as Explainable AI (XAI) has the objective of clarifying how predictions are made, offering an insight into the internal mechanisms of these algorithms [28].

There is a real need for effective Uncertainty Quantification (UQ) methods, which could incentivize clinicians’ confidence in and integration of AI models into clinical practice. UQ methods, both for epistemic and aleatoric uncertainties, are well established in computer science and are useful for the characterization of the limits of AI tools, but they are only in the early stages in their applications in healthcare. In the auto-segmentation field, methods such as conformal prediction can help in flagging cases with a low probability of correct segmentation and which need further human intervention [29].

Among the automated approaches, atlas-based segmentation methods have gained popularity in commercial systems. These methods typically involve selecting one or more pre-segmented atlases and deforming them to match the patient’s anatomy to generate contours. Various methods exist for selecting the best atlas or combination of atlases [25].

Studies have evaluated atlas-based auto-segmentation for different anatomical sites and structures, including head and neck OARs and prostate OARs. For example, research has assessed atlas-based auto-segmentation tools for the head and neck OARs, pre-clinically and clinically validating them for multiple target volumes and normal tissues like swallowing and mastication structures [30].

More recently, advances in machine learning, particularly deep learning, have led to the development of sophisticated automated segmentation and planning tools [26,30,31,32,33,34].

Deep learning algorithms, such as CNNs and Fully Convolutional Networks (FCNs), have shown promise in segmenting anatomical structures [30].

Examples include deep learning for the clinically applicable segmentation of head and neck anatomy and the automated CT segmentation of prostate cancer anatomy [31,35].

Traditionally, the auto-segmentation approach was based on intensity analysis (based on differences in imaging intensity among different tissues), shape modeling (based on the typical anatomical aspect of the structures of interest), and atlas-based techniques (based on a database of previously delineated structures), derived from retrospective peer reviewed treatment contours. They required substantial manual editing. The advent of deep learning models, especially CNNs, has led to a paradigm shift in auto-segmentation approaches. Indeed, they can handle a wider variety of complex anatomical structures; in particular, CNNs can extract hierarchical features from medical images through layers of learned convolutional filters [36].

Machine learning and AI applications are rapidly finding their way into the radiotherapy workflow.

Auto-segmentation solutions, particularly those leveraging deep learning, are being actively explored to alleviate these burdens, with the latter demonstrating improved accuracy over atlas-based techniques [31]. Despite this potential, the translation of deep learning-based auto-segmentation into routine clinical practice has been slow [3]. This delay in its adoption is likely associated with the current lack of comprehensive knowledge and standardized guidelines for the effective commissioning and implementation of these machine learning tools [3,32]. Moreover, this time-consuming and subjective task can lead to both inter and intra-variability between healthcare professionals.

Radici et al. reported time savings across multiple sites using Limbus Contour^®^, with the maximum advantages seen in head and neck cancer (65%-time savings), though time reductions for prostate (44%), breast (25%), and rectum (38%) have also been reported [37].

The recent emergence of AI used in medicine has also led to innovations in radiation oncology, offering significant advancements in treatment precision and workflow [38]. Recent advancements in AI have led to the development of auto-segmentation algorithms capable of delineating anatomical structures with a precision that rivals the expertise of human radiation oncologists. Watkins et al. explored the efficiency gains achievable with unedited AI-generated contours in total marrow irradiation, highlighting the potential of AI to achieve a 100% improvement in efficiency compared to traditional manual methods [39]. Another study reported that deep learning-based auto-segmentation reduces contouring time and improves clinical workflow efficiency in the treatment of cervical cancer [40]. A study focusing on the auto-segmentation of target volumes and OARs in pediatric cancer patients emphasized the importance of both accuracy and time efficiency in this vulnerable group. The study highlighted the potential of AI to strike a delicate balance between effective treatment and the preservation of normal tissue, which is crucial for minimizing potential growth-related complications in young patients [41]. Prior research suggests that AI can perform radiation contouring with a precision comparable to, if not exceeding, that of human oncologists. However, the strongest evidence of AI’s efficacy lies in its ability to replicate the nuanced expertise of human practitioners. In the AI conference co-hosted by the Embassy of the Republic of Korea to the UAE, the Department of Health Abu Dhabi, and G42 Healthcare, comparative analysis was conducted, during which human radiation oncologists and the AI software Limbus^®^ AI were tasked with contouring the axilla and internal mammary nodal chain from CT scans of a post-lumpectomy breast cancer patient. Only a small proportion of healthcare professionals and AI experts could correctly identify the AI-generated contour [42].

**Figure 1:**
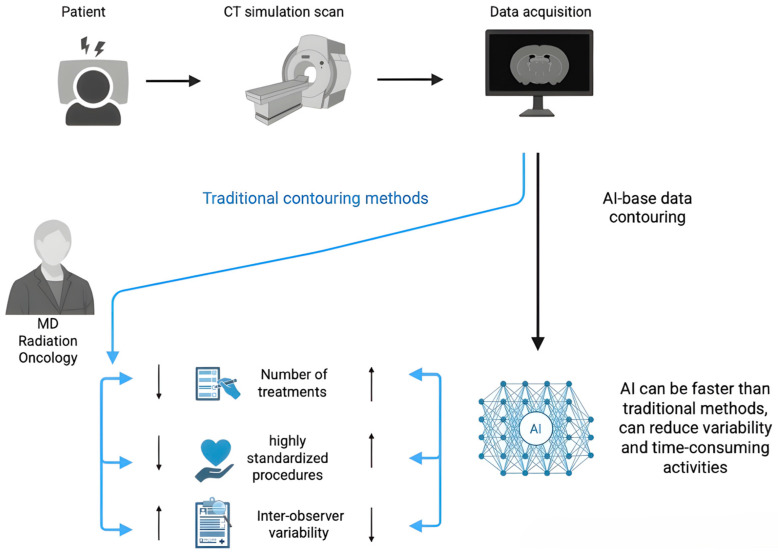
Comparison between traditional contouring methods used in radiation oncology and AI-based data contouring. Created in BioRender. Cristiano Grossi. (2025) https://app.biorender.com/illustrations/681bc74040787bb5138b50f9.

Auto-segmentation, facilitated by AI, holds promise for streamlining the manual contouring process. Studies suggest that AI can not only enhance efficiency but also reduce discrepancies arising from clinician interpretations [24,31]. Deep learning-based auto-segmented contours (DCs) have demonstrated remarkable accuracy, closely matching that of manual contours and surpassing that of traditional atlas-based methods [24,43] (Figure 1).

Despite the promising capabilities of AI-powered contouring, widespread clinical adoption remains limited [32]. Wong et al. reported the impact of DC models in the clinical workflow at two centers; in a previous study, they also conducted a comparison between DC and multiple radiation oncologists for central nervous system (CNS), head and neck (H&N), and prostate OARs and CTVs [24].

Further research is necessary to address the variability observed in the quality of DC models [33,44]. Rigorous studies are crucial to verify the reliability of specific models before their full integration into routine clinical practice [34]. This focus on robust validation will ensure the safe and effective implementation of AI in radiation oncology.

Seeking to streamline the radiotherapy workflow, in our institution, we investigated the clinical implementation of a commercial deep learning-based auto-contouring software. Wong et al. in 2020 reported on investigated CTVs in HN cancer and prostate gland cancer, but nonpelvic CTVs were included in their analysis [24].

Ethical concerns need to be considered in the development phase of AI tools. Ethical considerations in AI applications in healthcare call attention to issues such as security and privacy, the evaluation of AI models’ reliability, the appropriate settings of applications of AI models, the allocation of responsibilities and the need for human monitoring of the results obtained with AI processes. Instruments for systematic ethical assessments of generative AI in healthcare have been developed, such as the TREGAI checklist, based on nine generally recognized ethical principles [45].

In this study, we aimed to assess the software’s impact on contouring key pelvic structures, especially major pelvic lymph nodes, but also including the bowel, bladder, rectum, and sigmoid colon, within a homogeneous population with a diagnosis of PC. This study represents the first evaluation of major pelvic lymph node contouring, and may act as a valuable reference for other oncological diseases, including rectal cancer, anal canal cancer, and gynecological malignancies.

## 2. Materials and Methods

To streamline the radiotherapy workflow at our institutions, we investigated the clinical implementation of Limbus Contour software (Limbus AI Inc., Regina, SK, Canada, version 1.7.1) [46]. This study assessed the software’s impact on the contouring of key pelvic structures, including the bowel, the bladder, the rectum, the sigmoid colon, and major pelvic lymph nodes.

For this study, we investigated the consistency and the geometric reliability of LC in patients with PC and pelvic lymph node involvement. Our analysis deliberately excluded the prostate gland, because previous authors previously demonstrated the accuracy of this method for delineating the whole prostate gland [47]. Our focus was on structures located outside the prostate gland.

We chose to evaluate pelvic structures due to their high inter-patient variability. This characteristic makes them a good target for testing the software’s robustness in handling anatomical variations. Traditional segmentation methods, like atlas-based approaches, rely on pre-segmented image databases, which often require significant editing [25,43]. AI-powered contouring tools, like Limbus Contour, aim to overcome these limitations by offering quicker and more accurate delineations of target volumes and OARs. This has the potential to significantly improve patient outcomes through more precise radiation delivery [37].

We retrospectively analyzed data from 52 patients, treated at Mauriziano Umberto I Hospital, Turin, and “Umberto Parini” Hospital, Aosta, Italy, diagnosed with PC, comprising 40 patients with locally advanced PC and 12 patients with recurrent PC, treated between 2018 and 2024. All patients received either radical radiotherapy or adjuvant radiotherapy using the Volumetric Modulated Arc Therapy-Image Guided Radiotherapy (VMAT-IGRT) technique. All patients provided written informed consent approved by our Internal Institutional Review Board.

### 2.1. Software Description

In this study, we employed LC version 1.7.1, a commercially available auto-contouring software powered by deep learning algorithms. This advanced tool utilizes deep CNNs, with each anatomical structure being assigned a dedicated model specifically tailored for its unique features. These models are based on the widely adopted U-net architecture, a neural network design recognized for its effectiveness in biomedical image segmentation tasks [30,48,49].

The training process for LC models draws from an extensive and diverse array of imaging datasets. These include both publicly accessible datasets and proprietary collections obtained through collaborations with a range of medical institutions. Together, these sources form a robust and heterogeneous foundation for training the models, enhancing their generalizability and performance across different patient populations and imaging conditions [50,51,52,53,54,55,56,57,58,59]. The number of scans used to train each model typically ranges from several hundred to several thousand, ensuring that each model is supported by a substantial volume of learning data, which contributes to its accuracy and reliability.

To maintain high standards of performance and clinical relevance, Limbus AI implements a stringent dual-phase validation strategy. The first phase is an internal validation, where the software’s auto-generated contours are systematically compared against expert-drawn annotations on a designated test dataset. This process provides an initial benchmark of the model’s precision and segmentation accuracy. The second phase involves external validation through peer-reviewed publications, where independent studies assess both the qualitative and quantitative performance of the models. These studies also explore the software’s ability to enhance and expedite clinical workflows, thereby supporting its utility in real-world radiotherapy planning scenarios [24,50].

### 2.2. Contouring Process and Data Analysis

In this study, we assessed the level of concordance between organ delineations automatically generated by the Limbus AI software and those manually contoured by three experienced radiation oncologists using the RayStation System (RaySearch Laboratories, Stockolm, Sweden) as the treatment planning system (TPS), with the latest version available in 2024. The treatment plans and contours from our institutions were retrieved for analysis. The anatomical structures selected for delineation and analysis included key OARs and target volumes relevant to pelvic radiotherapy, specifically the bowel bag, bladder, rectum, and sigmoid colon, as well as the pelvic lymph nodes, which were contoured as part of the CTV. These structures were chosen due to their clinical importance in treatment planning and the potential variability in manual contouring. For each patient case, a duplicate set of the original manually defined structures was created within the Limbus software. These auto-generated contours were designed to mirror the original anatomical regions but were automatically labeled with a “Limbus” suffix. This labeling strategy was employed to clearly distinguish between the auto-contoured structures and those manually delineated by the radiation oncologists, ensuring clarity during subsequent analyses.

These new Regions of Interest (ROIs) were then exported back to RayStation for analysis using custom Python scripting (version 3.12) in terms of the following metrics:

1. Volume: The absolute volume of each ROI expressed in cubic centimeters (cc).
2. Dice Coefficient (DC): A measure of conformity, reflecting the spatial overlap between two delineated volumes. A value of 1 indicates perfect overlap.
3. Precision: The proportion of voxels identified by Limbus that truly belong to the OAR, reflecting the accuracy of inclusion. A value of 1 indicates only true positives (with no irrelevant structures included).
4. Sensitivity: The proportion of voxels in the true OAR that are correctly identified by Limbus, reflecting the completeness of delineation. A value of 1 indicates all true positives (no missed voxels).
5. Specificity: The proportion of voxels outside the true OAR that are correctly excluded by Limbus, reflecting the ability to avoid irrelevant structures. A value of 1 indicates only true negatives (with no false positives included).
6. Mean Distance to Agreement (Mean DA): The average distance between the surfaces of structures identified in both delineations and those identified by only one delineation. A value of 0 indicates perfect agreement in surface location.
7. Maximum Distance to Agreement (Max DA)**:** The largest distance between any corresponding surface points in the two delineations. A value of 0 indicates perfect spatial overlap.

The following sections will provide detailed explanations of each metric (precision, sensitivity, specificity, Mean DA, and Max DA) in the context of organ delineation and their clinical significance.

## 3. Results

Fifty-two patients were included in this retrospective analysis. Table 1 summarizes the dosimetric evaluation of organ delineation using all metrics listed previously.

CTV achieved good agreement with clinician contours, with a median Dice of 0.73 and a median volume difference of −56.82 cc. However, the model’s precision for CTV segmentation was lower (0.58), indicating a higher rate of false positives. In some cases, non-target structures like the bowel were included in the segmentation. The sensitivity was better than specificity (0.79 and 0.65, respectively). These findings suggest that, while the model can accurately delineate the CTV, in some cases, false structures were included in CTV, so further refinement is necessary to improve the precision of segmenting this structure. In contrast, delineation of the bowel bag using LC resulted in a lower median Dice coefficient (0.60) and a larger median volume difference −417.25 cc. This discrepancy likely arises from the excessively large LC margins incorporating the neighboring structures within the bag, as reflected by the lower precision (0.45) value for the bowel bag segmentation. Specificity reached an unexpectedly high value (0.83).

**Figure 2:**
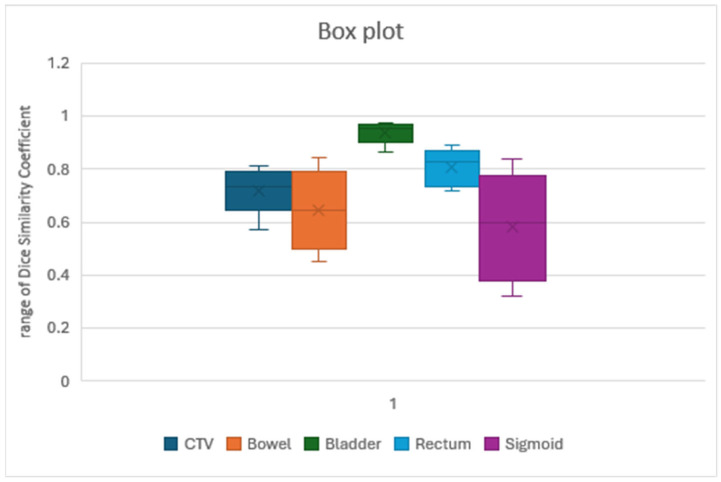
Variability range of Dice distribution for the CTV and OARs analyzed. The bladder achieved the best results, with the least differences, while the sigmoid had the worst results, with wide variability and the lowest median Dice. Similar results were obtained with the bowel. The rectum had excellent results. The CTV reached an acceptable value for a short while.

Bladder delineation achieved excellent agreement, with the highest median Dice (0.97) among the OARs evaluated (Figure 2). Volume differences were also minimal (median: −0.09 cc). A similarly excellent result was also obtained for precision, specificity, and sensitivity (0.94, 0.97, and 0.98, respectively). The mean DA achieved was 0.07, with a maximum DA of 0.66.

Similarly to the outcomes for the bowel bag segmentation, sigmoid delineation using LC proved challenging. The median Dice value for the sigmoid was 0.6, and the median volume difference was −13.68 cc. Precision was confirmed to be very poor, at 0.44, the worst of all analyzed structures. Similarly, the specificity was 0.41. We frequently observed inconsistencies between the LC-defined cranial margin of the sigmoid and the clinician’s delineation. In some cases, a gap existed between the sigmoid and the rectal Limbus Contour. In contrast, the rectum exhibited good agreement with a median Dice value of 0.76 and a negligible median volume difference, 3.05 cc. The precision value was 0.76, and the sensitivity was 0.86; therefore, LC did not miss many voxels.

## 4. Discussion

The present study investigated the clinical implementation of a commercial deep learning-based auto-contouring software (Limbus Contour, LC, version 1.8) for PC patients with pelvic lymph node involvement in a radiation therapy workflow.

**Figure 3:**
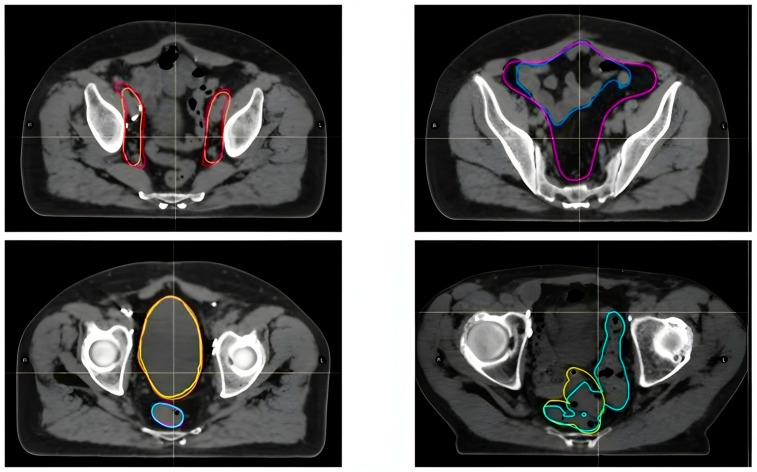
Orange CTV made by radiation oncologists, Red CTV made by Limbus; Blue Bowel made by radiation oncologists, Purple Bowel made by Limbus; Yellow Bladder made by radiation oncologists, Orange Bladder made by Limbus; Pink Rectum made by radiation oncologists, Light blue Rectum made by Limbus; Olive sigmoid made by radiation oncologists, Turquoise sigmoid made by Limbus. Orange line: CTV contoured by radiation oncologists. Red line: CTV contoured by LC. Blue bowel: bowel contoured by radiation oncologists. Purple bowel: bowel contoured by LC. Yellow bladder: contoured by radiation oncologists. Orange bladder: contoured by LC. Pink rectum: contoured by radiation oncologists. Light blue rectum: contoured by LC. Olive sigmoid: contoured by radiation oncologists. Turquoise sigmoid: contoured by LC.

The primary aim was to evaluate the software’s impact on the contouring of key pelvic structures, with a particular focus on major pelvic lymph nodes, alongside the bowel bag, bladder, rectum, and sigmoid colon (Figure 3).

To the best of our knowledge, this research represents the first evaluation of major pelvic lymph node contouring using an auto-contouring software, acting as a potentially valuable reference for other pelvic malignancies.

Previous studies have extensively evaluated the performance of auto-segmentation software, including deep learning models, for commonly contoured organs-at-risk (OARs) such as the bladder, rectum, and prostate, often demonstrating excellent results [37,60,61].

Van Dijk et al. classified vDSC (volumetric Dice similarity coefficient) scores into good (vDSC > 0.8), good–intermediate (0.7 < vDSC < 0.8), intermediate (0.6 < vDSC < 0.7), intermediate–poor (0.5 < vDSC < 0.6), and poor (vDSC < 0.5) [62]. However, it is important to remember that vDSC scores are relative; a score of 0.8 signifies high accuracy for small organs like optic nerves but indicates lower accuracy for larger structures such as the bowel or lungs. While we observed a good median Dice coefficient for the CTV (0.73), with the value generally considered clinically acceptable being around 0.72, according to previous studies [61], a major limitation exists. Unlike head and neck applications where lymph node sub-regions are identified and contoured separately, LC does not generate different structures for iliac, obturator, presacral, or inguinal lymph nodes and does not contour inguinal or lombo-aortic lymph nodes. This hinders its applicability in gynecological and gastrointestinal cancers, in which these important lymph node stations are absent. However, these settings are beyond the scope of our investigation.

Despite the strong performance of AI on many OARs, it is crucial to note that certain structures or specific regions often require significant manual editing. In a study by Cha et al. focusing on prostate Stereotactic Body Radiation Therapy (SBRT), 33% of the auto-contours required major, clinically significant edits based on physician surveys. While OARs generally required minimal to moderate changes, structures like the CTV in the pelvic region (specifically the prostate and seminal vesicles in the study mentioned) and the penile bulb had a greater necessity for significant modification [31].

Wong et al., evaluating deep learning-based auto-contours, reported that for bladder and rectum OARs, the average editing scores were 2 or less on a five-point scale (where 1 means minimal editing) [24].

Similarly, Zabel et al. compared manual contouring (MANpreRO) with atlas-based (ATLASpreRO) and deep learning-based (DEEPpreRO, using Limbus Contour) auto-contouring for the bladder and rectum in prostate cancer patients. Their findings indicated that DEEPpreRO contours showed greater geometric similarity to manual contours, with significantly higher DSC values and a lower Mean Surface Distance (MSS) for both structures [60].

Radici et al., using Limbus Contour for various sites, reported an average DSC of 0.72 across all analyzed structures [37].

The performance of LC varied across the different OARs, as can be seen in Figure 2. Bladder delineation achieved excellent agreement, with a high median Dice coefficient (0.97) and good precision (0.94), specificity (0.97), and sensitivity (0.98). This aligns with previous findings in several studies [60,63]; Kim et al. reported a lower Dice value, but they tested LC version 1.5 and 1.6 only on 10 pelvic patients [64]. In an earlier atlas-based study carried out in 2016, Wong et al. [65] reported median DSC values of 0.90 for the bladder, 0.77 for the rectum, and 0.84 for the prostate with the recommended settings. These values generally support the potential of deep learning to achieve high geometric similarity for common pelvic OARs.

However, bowel delineation presented challenges. The median Dice coefficient (0.62) was lower than that for the bladder, and the precision (0.45) was particularly poor, indicating the inclusion of irrelevant structures. This finding aligns with Radici et al. [37], who reported discrepancies between manual and automatic bowel segmentation. Doolan et al. analyzed only 20 prostate patients with five commercial AI auto-segmentation devices and reported a very low dice value (vDSC 0.59−0.76) for the bowels, but they did not test LC [66].

LC offers the opportunity to choose between single loops or whole-bowel delineation. In our study, whole-bowel volumes were significantly larger than manually contoured ones, potentially impacting treatment planning.

Although LC’s performance was evaluated for abdominal OARs in a recent study, the bowel was not included in our analysis [67].

Similar results were observed for the sigmoid. While the median Dice coefficient (0.6) suggests some overlap, the low precision (0.44) indicates the frequent inclusion of non-sigmoid structures. In further detail, discrepancies in manual and LC delineation of the rectosigmoid junction were frequently observed.

LC’s capabilities regarding rectum contouring were totally different. Our study reported a Dice coefficient of 0.87a, value close to that (0.86) reported in previous research by Zabel et al. [60] and Oktay et al. (0.90) [63], indicating good overlap with manual contours. However, our study suggests that LC may exhibit greater difficulty with sigmoid contouring compared to contouring of the rectum, a limitation seen with many other AI auto-segmentation softwares [66]. This aligns with our findings of a lower Dice coefficient and lower precision for the sigmoid.

Despite the difficulties in precisely quantifying the time savings, perceived utility and observed improvements in workflow efficiency are frequently reported. Zabel et al. found that deep learning auto-contouring (using Limbus Contour) for the bladder and rectum significantly reduced the initial contour generation time (1.4 min for DEEP vs. 10.9 min for manual) without significantly increasing the time required for physician editing compared to manual methods (4.7 min for DEEP editing vs. 4.1 min for manual editing) [61].

This study has limitations, including its retrospective design and limited sample size. Further research with a larger and more diverse patient population is necessary for generalizability. Future studies should investigate the potential of AI algorithms to accurately delineate not only pelvic lymph nodes in various cancer types, but also the bowel and sigmoid. This could significantly improve the efficiency of radiotherapy planning and reduce the workload for radiation oncologists.

Another limitation is the lack of a dedicated dosimetric analysis to assess the impact of using auto-contours instead of manual contours in terms of DVH metrics.

The most recent version of LC, version 1.8, can contour new structures for example inguinal lymph nodes station, but it cannot contour other structures for example lombo-aortic lymph nodes yet. Furthermore, differentiation of pelvic lymph nodes (iliac, obturator, and presacral), such as those in the head and neck cancer setting, is not permitted.

Overall, this study provides valuable insights into the potential and limitations of using LC as a supporting instrument for pelvic structure delineation in radiotherapy. Accurate comparison with studies in the previously published literature is inherently difficult. This is due to variations in the definition of “gold-standard” manual contours, differences in arbitration and consensus-building processes, variations in the datasets used (including patient demographics, disease sites, stages, and tumor types), diverse scanning parameters and image devices, and different labeling protocols [30].

Quantifying time savings accurately in a real-world clinical setting remains challenging.

## 5. Conclusions

In conclusion, the findings from this study, supported by the growing body of evidence in existing research, demonstrate the capability of deep learning approaches to achieve high-quality auto-segmentation for radiotherapy, often reaching performance levels comparable to that of human experts for common OARs. The evaluation of Limbus Contour, particularly for pelvic structures, confirms its potential utility in the clinical workflow by reducing the initial contouring burden and providing suitable starting points for required edits. While significant progress has been made, particularly for well-defined OARs, challenges remain in achieving consistent accuracy for all structures and in precisely quantifying the impact on clinical workflow efficiency. Continued refinement of AI models, addressing areas of frequent editing, establishing clinical consensus on contouring protocols, and providing adequate training and robust QA frameworks are necessary steps to maximize the benefits of auto-segmentation in radiotherapy and improve consistency and safety in clinical practice. These challenges require further development and validation before AI’s broader clinical adoption.

By overcoming these limitations and exploring LC’s applicability across diverse cancer types, we can significantly improve the efficiency and precision of treatment planning in the future. Ultimately, this translates to better patient outcomes in cancer care. Continued advancements in AI-driven technologies hold immense potential for optimizing radiotherapy.

## Figures and Tables

**Table 1:** The median average of all dosimetric data evaluated for each structure.

	CTV	Bowel	Bladder	Rectum	Sigmoid
**Diff. Vol (cc)**	−56.82 (−205.96; 185.94)	−417.25 (−1500.57; 5168)	−0.09 (−22.27; 14.58)	3.05 (−18.91; 23.73)	−13.68 (−198.91; 82.34)
**Dice**	0.73 (0.53; 0.84)	0.62 (0.36; 1)	0.97 (0.77; 1)	0.87 (0.57; 1)	0.60 (0.2; 1)
**Precision**	0.58 (0.36; 0.73)	0.45 (0.22; 1)	0.94 (0.63; 1)	0.76 (0.4; 1)	0.44 (0.11; 1)
**Sensitivity**	0.79 (0.57; 0.97)	0.65 (0.24; 1)	0.97 (0.76; 1)	0.86 (0.61; 1)	0.85 (0.22; 1)
**Specificity**	0.65 (−0.2; 0.96)	0.83 (−1.08; 1)	0.98 (0.72; 1)	0.91 (0.09; 1)	0.41 (−6.93; 1)
**Mean DA**	0.39 (0.18; 0.97)	1.2 (0.01; 3.98)	0.07 (0; 0.35)	0.15 (0; 1)	0.73 (0; 3.43)
**Max DA**	3.25 (1.76; 6.65)	8.39 (0.02; 16.51)	0.66 (0.01; 2.26)	1.3 (0.01; 5.52)	5.44 (0.01; 17.09)

## Data Availability

The data presented in this study are available on request from the corresponding author.

## Abbreviations

The following abbreviations are used in this manuscript:

RT	Radiotherapy
PC	Prostate Cancer
OARs	Organs at risk
LC	Limbus contour
RP	Radical Prostatectomy
BCR	Biochemical Recurrence
ART	Adjuvant Radiotherapy
GS	Gleason Score
SRT	Salvage Radiotherapy
PNRT	Pelvic Nodal Radiotherapy
ENRT	Elective Nodal Radiotherapy
CT	Computed Tomography
CTV	Clinical target volume
AI	Artificial Intelligence
DVHs	Dose Volume Histograms
CNNs	Convolutional Neural Networks
XAI	Explainable AI
UQ	Uncertainty Quantification
FCNs	Fully Convolutional Networks
DCs	Deep Learning-Based Auto-Segmented Contours
CNS	Central Nervous System
H&N	Head and Neck
VMAT-IGRT	Volumetric Modulated Arc Therapy-Image Guided Radiotherapy
TPS	Treatment Planning System
ROIs	Regions Of Interest
cc	Cubic Centimeters
DC	Dice Coefficient
DA	Distance to Agreement
vDSC	Volumetric Dice Similarity Coefficient
SBRT	Stereotactic Body Radiation Therapy
DEEP	Deep learning auto contour
